# Supplementary material for: Development of a small and sick newborn clinical audit tool and its implementation guide using a human-centred design approach newborn clinical audit process and design
Source: PLOS Glob Public Health. 2023 Feb 23;3(2):e0001577. doi: 10.1371/journal.pgph.0001577 (PMC10021839; doi:10.1371/journal.pgph.0001577)
Supplement: S1 Appendix — (DOCX) [file pgph.0001577.s002.docx]

| **Supplementary file 1 - Description of consensus workshop to develop a SSNB audit implementation guide** |
| --- |
| One day virtual workshop with 37 purposely selected neonatologists, NBU nurse leaders, paediatricians and an MoH official (2 neonatologists, 17 paediatricians, 17 NBU nurse leaders and 1 representative from the Ministry of Health) held using the Zoom platform and aiming to answer:   1. What are the facilitators and barriers to the SSNB audit process in the Kenyan context? 2. How can we design the implementation guide to build on the strengths and overcome the barriers? 3. We conducted FGDs with the 37 workshop participants. A purposive sampling strategy was used to divide the participants into four groups using Zoom breakout rooms with a sample size of 9-10 each. The purposive sampling ensured there was an equal number of nurses and paediatricians in each group. Each of the four groups had: 4. A moderator who was either a nurse or a paediatrician who had group facilitation skills as they had gone through the UK Resuscitation’s Council Generic Instructor’s Course (GIC) in Kenya.[29] 5. A rapporteur who was a researcher from KWTRP who had experience in conducting qualitative research and moderating small groups. 6. A note taker who was a paediatric resident from UoN, was GIC trained and was proficient in using the Zoom platform and its different features. The rapporteur helped to manage group dynamics and the note taker helped to document responses on the virtual whiteboard.   Each group moderator had a discussion guide with open-ended questions to help structure the discussions and each group had a different set of questions. The discussion guide also contained probing questions to help the moderator cover issues in depth with the less talkative groups. Consent to participate in the FGDs was obtained through a two-step process; i) An electronic version of the consent forms was emailed and sent via WhatsApp to each invited participant three days before the workshop. They were requested to sign the form or fill in their names and send it back to the main facilitator (MO) before the workshop if they agreed to participate in the workshop, ii) before beginning the FGDs, each moderator projected the consent form and a confidentiality agreement statement and read them out to the group members. The participants were then asked to directly message MO via Zoom chat with the statement “I agree” or unmute and confirm that they agree if they agreed to participate. Data collection was done through audio recording as well as the information documented on the whiteboard.  **Data analysis**  The data were managed on NVivo 12 software. The data analysis was done through thematic analysis. The audio-recorded data from the FGDs were transcribed verbatim. After transcription, the first step was familiarisation with the data by reading through it severally. Two researchers, MO and GI independently studied the transcripts and independently generated the initial codes. An abstraction process then took place and the codes were grouped into themes. The entire analytic process involved collaboration with MO and GI, and all discrepancies were discussed until consensus was reached.   1. Interactive one-hour plenary session with discussions on the audit implementation guidelines for LMICs based on the recommendations Geneva: World Health Organisation; 2018 manual. The discussion focused on:  - Defining a clinical audit process. - Describing the six steps of the audit cycle as per the WHO guidelines (1. Identifying a case for audit, 2. Collection of information, 3. Identifying cause of death and modifiable factors, 4. Recommending solutions, 5. Implementing recommendations ,and 6. Monitoring and evaluation) with emphasis on modifiable factors and the importance of completing the audit cycle. - Describing the components of a quality audit process based on the Geneva: World Health Organisation; 2018 manual e.g. presence of a multidisciplinary team, creating a conducive environment for the audit meeting, scheduling of meetings and maintaining confidentiality. - Introducing the prototype newborn audit tool and explaining how the audit tool is intended for use during the audit meetings.  1. Adaptation by consensus on a context sensitive implementation guide for the SSNB audit process that took place in 2 steps:  - Creation of user personas which are fictional characters that represent the different users of the implementation guide (e.g. nurses, paediatricians, nutritionists, medical officers). The process enabled the participants gain insight into the needs, behaviours, experiences and goals of the different users and therefore understanding their requirements for the successful uptake of the implementation guide. - With deeper understanding of the users and their requirements, the participants arrived at consensus on the audit implementation guide using a modified nominal group technique. This method was selected because it allows for prioritisation of thoughts and ideas, encourages equal participation among members of the group and allows for quick problem solving. Throughout the group consensus process, participants were given space to think about the identified problem, make individual decisions and these were recorded through individual voting and tallying on the virtual whiteboard. |
