# Supplementary material for: Development of a small and sick newborn clinical audit tool and its implementation guide using a human-centred design approach newborn clinical audit process and design
Source: PLOS Glob Public Health. 2023 Feb 23;3(2):e0001577. doi: 10.1371/journal.pgph.0001577 (PMC10021839; doi:10.1371/journal.pgph.0001577)
Supplement: S2 Appendix — (PDF) [file pgph.0001577.s003.pdf]

Remove Watermark Now

Consent Form for Focus Group Discussion on the Facilitators and Barriers to the Newborn Unit Quality of Care Audit Process in County Hospitals in Kenya

Introduction

My name is Dr. Muthoni Ogola and I am currently studying for a PhD at the University of Nairobi. I am conducting a research study on the impact of the newborn unit audit process on identifying modifiable factors in preterm feeding practices. I am going to give you information on the study and then invite you to be part of this research.

A well conducted audit process is a quality improvement initiative that aims to identify the modifiable factors in patient care by determining if the care provided was consistent with evidence-based guidelines. My work focuses on designing and implementing a standardised newborn audit process in County Hospitals in Kenya.

pdfelement

Type of research intervention

Newborn unit health care workers will respond to this interview guide to help identify the facilitators and barriers to the newborn audit process in their hospitals.

Study procedure and duration

If you agree to participate in the study, I will invite you to respond to these questions that seek to **identify the facilitators and barriers to conducting the newborn audit process** in the County hospitals. I will not indicate any participant or hospital names or details.

Risks

There are no risks to participating in this study as no identification information will be used and everything discussed will be confidential. We will strictly discuss facilitators and barriers within the system and not individual people.

Remove Watermark Now

Benefits

There are no individual benefits to participating in the study, however, this will enable the research team to understand the strengths and weaknesses within the health facilities that influence the success of the newborn audit process.

Re-imbursement

There will be no re-imbursement for responding to this interview guide.

Confidentiality

Hospital identification will be coded. I am the only person who will know which hospital the codes belong to.

Sharing of results

We will conduct a feedback forum for newborn unit paediatricians and nurse leaders at the end of the study to provide feedback on our study results. At the end of the study, we will also publish the results so that other interested people may learn from our research. The information that will be provided to the hospital team and published will not have any identifiers that can be linked back to you.

Right to Refuse or Withdraw

Your participation in this research is entirely voluntary. It is your choice whether to participate or not.

You do not have to take part in this research if you do not wish to do so. You can also withdraw at any time if you feel uncomfortable to continue with the interview. Refusing to participate will not affect you or your hospital in any way.

Who to Contact?

If you have any questions you may ask them now or later, even after the study has started. If you wish to ask questions later, you may contact me using this address or telephone number.

pdfelement

Remove Watermark Now

Muthoni Ogola

MOgola@kemri-wellcome.org

Telephone No: 0722435015

You may also contact my supervisors using these contacts:

Prof. Grace Irimu,

Email: GIrimu@kemri-wellcome.org

Telephone number: 0722564600

Prof. Mike English

Email: MEnglish@kemri-wellcome.org

Dr. Jalemba Aluvaala

Email: JAluvaala@kemri-wellcome.org

Telephone number: 0722217034

Ethical approval for the research has been covered under: A System Strategy to Optimise Neonatal Inpatient Care in Kenyan Hospitals (Sonic Study) - KEMRI/SERU/CGMR-C/161/3852.

pdfelement

PART II: Certificate of Consent

I have read the foregoing information, or it has been read to me. I have had the opportunity to ask questions about it and any questions that I have asked, have been answered to my satisfaction. I consent voluntarily to participate in this research.

Name of participant ______________________________________

Signature of participant ______________________________________

Name of researcher ______________________________________

Signature of researcher ______________________________________

Remove Watermark Now
