# Supplementary material for: Development of a small and sick newborn clinical audit tool and its implementation guide using a human-centred design approach newborn clinical audit process and design
Source: PLOS Glob Public Health. 2023 Feb 23;3(2):e0001577. doi: 10.1371/journal.pgph.0001577 (PMC10021839; doi:10.1371/journal.pgph.0001577)
Supplement: S4 Appendix — (DOCX) [file pgph.0001577.s005.docx]

Table 4: Categorisation of Identified Changes to the Prototype Audit Tool Based on Usability, Human Factors and User Experience and the Outcomes of the Feedback

| **Audit tool number** | **Usability** | **Human Factors** | **User experience** | **Outcome** |
| --- | --- | --- | --- | --- |
| - Audit tool # 0 |  | - To reduce the length of the audit tool while maintaining important information. - To get details on section one; newborn and mother’s biodata and details of labour and delivery as a print-out from the CIN-Neonatal data clerks in the hospitals therefore reducing the workload of the clinicians filling the audit tool before the meeting. |  | - The 1^st^ page on newborn and mother's details was separated from the audit tool. - Content of the audit tool remained the same, but the structure changed such that the left half of the page was for documenting the summary of the care provided before the audit meeting and the right half was for documenting modifiable gaps identified in each section during the audit meeting and the recommendations made. |
| - Audit tool # 1 |  | - To structure the section on newborn and mother’s details as textboxes to make the tool easier to fill out. - To add more colour to the audit tool and make it neater. | - The audit tool seemed incomplete without the front page that had sections on newborn and mother’s biodata. - An observation that it was cumbersome to look at the coded section on maternal complications which was at the end of the audit tool. | - Returned section one on newborn and mother’s details. - Improvement on the aesthetics of the audit tool. - Format of the section on maternal complications was changed from selecting from a coded list to providing a free space for the clinicians to list any complications that may have been present. - The pages of the audit tool were divided into 3 sections. Section A as the summary filled before the audit meeting took 1/3^rd^ of the page. Section B took 2/3^rd^ of the page and had 2 parts – the 1^st^ was to document the modifiable factors arising from the audit meeting and the 2^nd^ was to document recommendations made during discussions. - The structure of the section on danger signs at admission was broken down to identify the specific abnormality in the vital signs e.g. if they were too high or too low as well as some specific danger signs that were considered critical. |
| - Audit tool # 2 | - To borrow from the neonatal admission record (NAR) form on the structure of the admission details as it is comprehensive and has the relevant information required during the audit. | - To make as many sections with checkboxes to reduce the workload for the clinicians filling the audit tool. | - To include a section on the neonate’s progress of illness after admission as there was a gap in the flow of information after admission details. | - Section on mother’s antenatal history and labour and delivery complications were designed with checkboxes and textboxes borrowing from the neonatal admission record (NAR) form. - Section on labour and delivery complications; we included a checkbox on whether the newborn was resuscitated at birth. - Section on danger signs and symptoms at admission was reformatted to include those in the NAR form. - Separate the sections on supportive and definitive management at admission.   We included a section for the clinician to document a summary of the progression of the child’s illness post-admission. |
| - Audit tool # 3 |  |  | - There was too much free text in the section on supportive management making it time-consuming to fill the audit tool. - The section on investigations was cumbersome and required to be structured in a way in which the important investigations are listed making it easier to fill. | - Section on investigations was structured further with each of the critical investigations listed and space provided to document the investigation results. - We included documentation of post-admission weights to the feed and fluid section. - The section on supportive management was further refined to the management of specific conditions that were considered critical. |
| - Audit tool # 4 | - No objective way of determining if the danger signs were recognized. - No provision to document the modifiable factors. | - Feedback to add more sections with checkboxes to reduce the workload for the clinicians filling the audit tool. | - The section required to determine if there was a delay in review at admission was too subjective and needed to be restructured. - To increase the writing space for sections with free text as the space provided was inadequate to provide relevant details. | - The structure of the audit tool was changed such that the pages were no longer divided into two parts, instead, the section for discussion came below the summary of care in each section. - In the section on review of care, we structured it to document time of birth, time of admission by NBU nurse and time of admission by the clinician in NBU. - We included a section on response to danger signs and symptoms at admission with checkboxes on the acceptable responses to danger signs based on the protocols. - The section on the critical basic laboratory and radiological investigations was formatted into a table that allowed for the documentation of each investigation result and the dates when the investigations were conducted. - The section on definitive management was further modified into a check box of the essential newborn medication based on the BPP. We also included columns to document the prescribed dosage, route and number of days the medication was prescribed. - We included a section at the end of the audit tool to document the modifiable factors identified from the discussion. |
| - Audit tool # 5 | - We presented the audit tool to neonatologists from KNH who suggested that we include a section to audit the management provided at referring facilities as a significant percentage of the newborn population in the NBU was referred in from a different facility and it was important to know the quality of care that was provided at those facilities. - Suggestion that we should not only audit delays in conducting investigations but also delays in acting for abnormal results. |  | - Feedback that we should group the audit tool into two sections.  1. The first section to be the summary of the case being audited (section filled before the audit meeting) 2. The discussion section (section filled during the audit meeting).   The experience was that discussing the quality of care after each section made the audit meeting take too long. | - The structure of the audit tool was changed to separate section A (summary of care provided) and section B (section for discussion of the quality of care during the audit meeting). We had section A as the 1^st^ part of the audit tool and section B as the 2^nd^ part. - Inclusion of details of referral for the patients referred into the newborn unit from different facilities. - Two columns were included in the table of investigations – a column on the action taken and a column on the date the action was taken. - Section on supportive management was modified to include the possible management options for each sign and symptom based on the basic paediatric protocols. |
| - Audit tool # 6 | - We included a section to document any medication that may have been used during the initial resuscitation after birth. This was in an attempt to highlight wrong practices during resuscitation. |  |  | - Section on newborn details – we increased the Apgar Score options to 20 minutes. - Section on newborn resuscitation after birth, we included options for any medication used during resuscitation and checkboxes for oxygen support post-resuscitation. - Section on investigations was restructured to the specific investigations and the dates the investigations were ordered, dates results were received, actions taken and the dates the actions were taken. |
| - Audit tool # 7 | - The team made observations that the audit process seemed like it was only auditing the care provided by the clinicians and the nursing care was left out. - Poor participation by nurses in the audit meetings. *“I don’t understand how you will find all cadres in the audit meeting; doctors, midwives, lab, pharmacy, you name it. But the nurses from the newborn unit never attend the meetings and when asked are always too busy.”* (Audit meeting participant) |  |  | - We added a section on vital signs monitoring for each day of life for the newborn. - We included columns on feed and fluid monitoring to the section on feed and fluid management. |
| - Audit tool # 8 | - The audit tool only allowed for the identification of the cases that were referred in and the reason given for referral. It however did not allow for details of the care provided at the referring facility. It was therefore not possible to identify gaps in care at the referring facilities. |  | - The section on response to danger signs at admission was repetitive. | - Audit tool was converted to an electronic tool using the application PDF element. - We deleted the section on response to danger signs at admission. - Included a section to summarise management at the referring facility if the case was a referral. - We planned to introduce the audit process to all the CIN-N sites and therefore transformed it into an electronic tool (E tool) as this would make it possible for the hospitals to adhere to the MoH regulations that prevented large meetings due to the COVID-19 pandemic by conducting virtual audit meetings. This would also allow us to attend the meetings virtually due to travel restrictions across counties. |
| - Audit tool # 9 |  | - To make it easier and faster to fill in the audit tool by adding drop-down calendars and checkboxes where possible. | - The end users had challenges in filling the section on modifiable factors as evidenced by the difficulty in filling the section during audit meetings. | - We changed the application used to design the audit tool from PDF Element to Adobe Acrobat Pro. - We included drop-down calendars to all the sections where a date was to be documented. - The modifiable factors were presented as a list as agreed upon during the consensus workshop. |
| - Audit tool # 10 |  | - Section on modifiable factors could still be made easier to fill. |  | - The list of modifiable factors was converted to a drop-down list under the major categories and sub-categories. |
| - Audit tool # 11 |  | - Feedback from the clinicians that a checkbox for the modifiable factors would be easier than the drop-down list. |  | - The format for the modifiable factors was changed from a drop-down list to a checkbox list. |
| - Audit tool # 12 | - The section on the medication used during resuscitation was misleading as it gave the impression that this was the proper management. Suggestion that we should instead promote the correct practice by focusing on bag valve and mask (BVM) and chest compressions. Any treatment beyond this should be put under others. |  |  | - We modified the section on newborn resuscitation after birth. We deleted the checkboxes on the medication used and included a checkbox on chest compressions. |
| - Audit tool # 13 |  |  | - To include the action plan summary form as part of the audit tool as it was frequently forgotten. | - We included the action plan summary form as the last section of the audit tool. |
| - Audit tool # 14 | - To include measurements of head circumference and length in the newborn admission examination details. |  |  | - We included the options of head circumference and length in the section on newborn details. - We modified section 3 on the review of the care provided. We included the date of admission by nurse and the date of admission by the clinician. S3 Appendix |

Abbreviations: CIN – Neonatal, Clinical Information Network for newborns; NAR, Newborn Admission Record form; NBU, Newborn Unit; KNH, Kenyatta National Hospital.

Definitions: Usability, “extent to which a user can use the audit tool to achieve specific goals with effectiveness, efficiency and satisfaction in a specified context”; Human Factors, “application of knowledge about human capabilities and limitations to the development of the audit tool”; User experience, “perceptions and responses of users that result from their experience of using the audit tool.” Danger signs – Abnormal vital signs (hypo/hyperthermia, brady/tachypnoea, brady/tachycardia, hypoxia, hypo/hyperglycaemia), general examination (dehydration, jaundice, pallor), respiratory system (apnoea, difficulty breathing), central nervous system (convulsions, difficulty feeding, reduced/absent movement), gastrointestinal system (bilious vomiting)
