# Supplementary material for: Development of a small and sick newborn clinical audit tool and its implementation guide using a human-centred design approach newborn clinical audit process and design
Source: PLOS Glob Public Health. 2023 Feb 23;3(2):e0001577. doi: 10.1371/journal.pgph.0001577 (PMC10021839; doi:10.1371/journal.pgph.0001577)
Supplement: S5 Appendix — (DOCX) [file pgph.0001577.s006.docx]

| **Themes** | **Sub-themes** | **Sub- sub-themes** |
| --- | --- | --- |
| **Facilitators** | | |
| 1. **Patient safety culture**   Patient safety culture is an integrated pattern of individual and organizational behaviour that continuously seeks to minimize patient harm that may occur from the care delivery process.[38] The study participants elaborated on the ways in which their organisations prioritized patient safety by viewing patient care from a systems perspective and therefore recognizing the value of creating an equal environment that supports open dialogue and an environment that encourages learning from preventable adverse events. | 1. ***Collaborative approach to patient care***   While some participants claimed that newborn audit meetings were only attended by the newborn unit health workers, others reported a different experience. The study participants expressed that the audit meetings provided an opportunity for different health worker cadres to work together and share responsibility for problem-solving and decision making. They pointed out that this team collaboration was important as a joint effort from a team with diversity in knowledge and skills would lead to solutions that best address the emerging avoidable gaps. The participants reported that the maternity/labour ward team were usually invited to the audit meetings, while the other cadres were invited based on the significance their input would add on a case to case basis.  ***“Yes, at our facility, we don't have a structured newborn audit committee, but as a department led by the paediatrician and the head of the department, we have monthly… monthly audit meetings in which we have a variety of people from different departments. We always make sure there is somebody from the laboratory, pharmacy, public health and the maternity.” FGD 1 (R2)***   1. ***Equality during audit meetings***   The study participants expressed that a non-hierarchical environment where every meeting participant was viewed as an equal and all contributions were respected facilitated a non-threatening environment.  ***“And audit meetings actually brought this out. It doesn't matter who you are, whether you are a professor, whether you're a paediatrician, whether I'm a nurse. You know...So we're all given, from experience, we were all given an equal opportunity to speak out our mind.” FGD 3 (R3)***   1. ***Learning from errors***   A strong patient safety culture encourages learning from modifiable factors which are preventable adverse events. The study participants agreed that the reflective nature of the clinical audit enables practitioners to draw inferences from their own practice by analysing what was done and interpreting the relationship between their practice and patient outcomes. This exposed the gaps in knowledge and skills and the participants therefore perceived it as a way to identify learning needs.  ***“In our case, in case we identify any mistake during our audit, we use it as a learning point. So, what we do we'll go over the condition the patient had and discuss it and help everyone to understand what we're supposed to do. It actually makes a topic for the next CME.” FGD 3 (R5)***   1. ***Frequent structured meetings***   A patient safety culture encourages routine quality improvement meetings that are well structured with the day, date and timing of meetings well known by the teams. This helps to maximise meeting attendance. Several of the participants reported that the meetings were held monthly.  ***“So basically, at H1, what we do is we just discuss the mortality. We do monthly audits. So we discuss the mortality and morbidity, individual cases. Uh, yes, we do individual cases. Ummm... The first Friday of the month.” FGD 2 (R2)***  In addition to ensuring that the meetings are well structured, several hospitals have adapted to using flexible means of communication to maximize audit meeting attendance.  ***“So, like for the written memos, to me I find that there's usually a bit of delay, and when you do when you do SMSs and emails, the outcome tends to be much better as compared to the written memos. So I would prefer doing the emails and the SMSs and preferably the WhatsApp messages of which the turnout becomes better than the written memos.” FGD 1 (R4)*** | ***Mentorship***  The study participants acknowledged the connectedness between the County hospitals and the lower level referring facilities. They recognised that the quality of care provided to patients in the lower level facilities before referral had a significant impact on the patient outcomes. Based on this, the FGD participants reported that they share knowledge on lessons learned during audit meetings with health workers from the referring facilities. They reported that the knowledge transfer is usually done through inviting these health workers to the County hospitals for a period to work under supportive supervision until they acquired the necessary skills.  ***“Hallo everyone, the only thing I would like to add is that, when we do the auditing, we are able to identify the gaps, and, recently we've been able to mentor our referring facilities, especially the nurses from the referring facilities. So they have come in the newborn unit for at least a week, so that we mentor them on how to how to manage the newborn units...newborns, especially when they are delivered having birth asphyxias, and how they would be referring them to our facilities, including doing Kangaroo Mother Care during the referral, during when they are referring the babies.” FGD 4 (R7)*** |
| 1. **Completion of the audit cycle**   An audit with no action will not lead to change. Poor implementation of audit identified recommendations has been identified as a major contributor to the loss of confidence in the audit process.[34, 35, 37] The study participants described the different strategies that they have put in place to ensure that recommendations are implemented. The strategies reported included; 1) direct task allocation, and 2) interlinked task-oriented minutes. | 1. **Direct task allocation**   Some study participants reported that to ensure accountability, each action plan arising from the audit meetings is assigned to a specific person in the relevant department.  This key person was responsible for ensuring that the action plan was implemented.  ***“So, we sat down as a department, everybody had an individual work plan. Because most of the recommendations we made are supposed to be implemented by the facility, and it's been taking long. So, we decided as a department, everybody will have an objective. So, we made an individual work plan, was it last month? Everybody is working on like... Everybody has an objective to reduce the number of deaths... like, all the nurses in the department, everybody has an objective. An example is somebody has an objective on resuscitation, another one on infection prevention” FGD 4 (R8)***   1. **Interlinked task-oriented minutes**   Study participants reported that one of the conditions that enforced implementation of recommendations from the audits was through taking minutes that focused on the identified modifiable gaps and the recommended solutions. The meetings are then interlinked by ensuring that one meeting is a continuation of the previous one through beginning each meeting by reading the minutes from the previous meeting and determining if the recommendations have been implemented.  ***“So that when you're coming in the next auditing day, we have now to look at the recommendations we have done, where have we reached? And, where are we?” FGD 4 (R7)*** |  |
| **Barriers** | | |
| 1. **Unhealthy organisational culture**   This main theme reflects the perceptions of the FGD participants with regards to the impact their organizational culture had on the effectiveness of the audit process. The emerging categories were: Limited support from hospital leadership, hierarchical relationships, name and blame culture and blame shifting. | 1. **Limited support from hospital leadership**   The respondents perceived the leadership style as an important determinant of the success of the audit process. There was general agreement among the health care workers that the senior hospital leadership had a more laissez-faire approach towards the audit process.[39] This was evidenced by their absence from audit meetings; leaving them to be conducted and managed by the mid-level managers. The study participants pointed out that they occasionally had to persuade the hospital leadership to attend when they required extra support to implement action plans beyond their control.  ***“Yes, if there is a recurring problem that we have identified that involves a particular team or player, say, for example, administrative matters. That is when we go ahead, we also seek the audience for the participation of the hospital leadership mainly by either the hospital CEO, the administrator or the nursing officer.” FGD 1 (R6)***   1. **Hierarchical relationships**   The study participants recognised that the relationships between the health worker cadres was hierarchical in nature. There was consensus that among the mid-level managers, the consultant was at the top of the hierarchical pyramid. The consultants independently determined if and when meetings would happen, who would be invited to the meetings as well as had the responsibility of chairing the meetings.  ***“most of the thing is the paediatrician who determines who comes into the meeting and also the date, the exact date when the meeting is on” FGD 1 (R6)***  The responses revealed that there was skewed task shifting such that when the consultant was not available, the responsibilities were automatically transferred to the junior doctor despite there being more senior representation from other cadres such as the nurses.  ***“That is not right. When the paediatrician is not there, we do delegate. Cause there are times when the paediatrician is out in other meetings... and... uh... usually like the paediatrician might have gone on leave. So, if there's a medical officer within that department, we usually like delegate that to the medical officer to run the audit meetings.” FGD 1 (R4)***   1. **Name and blame environment**   Participants described how one of the most significant deterrents to the success of the clinical audits was a culture where the audits were used as a as a ‘witch hunt’ rather than a quality improvement exercise. This instilled a fear of punishment and victimization among the health workers and therefore reluctance to participate in audit meetings.  ***“Labour ward you were supposed to do this, and it was not done, no, newborn you were supposed to do this, and it was not done. So, what I've realized, ok that is me, what I've realised, when there's an audit, in fact, I've realised that some people do not like attending because of that blame.” FGD 3 (R4)***   1. **Blame shifting**   We noted that there was a general belief among the respondents that the lower level referring health facilities were the major contributors to the newborn morbidity and mortality burden in their hospitals. The attitude among the FGD participants was that the newborn outcomes would greatly improve if the referring facilities improved the care they provided. This is despite data from the CIN for newborns showing that majority of the newborn deaths in the newborn units of these County hospitals were from babies born in their hospitals.[16]  ***“Yes, yes daktari and I like audits. But what I what I don't like is when we are discussing, like, cases of mortality due to birth asphyxias from referral facilities, and they are not there. You know, we… we… we… discuss things that we can't change as in the newborn unit. And then, when… when… we send recommendations to them, they do nothing, it's like they don't feel it.” FGD 4 (R6)*** |  |
| 1. **Health workers’ perceptions about the value of clinical audits**   The FGD participants revealed that the health workers did not fully appreciate the benefits of the clinical audit process on quality improvement. | 1. **Poor ownership of the audit process**   The MPDSR guidelines recommend that perinatal deaths are a notifiable event and that all the perinatal deaths should be reviewed at the facility level during monthly facility MPDSR meetings.[9] Based on the responses from the participants, it emerged that in some Counties, the clinical audits were not held at the hospital level. The health workers instead waited for the three-monthly Sub-county meeting to be convened in which every facility within the Sub-county only had a chance to present their morbidity and mortality statistics. We got the sense that the health workers did not own the MPDSR process as they did not appreciate the value conducting the audit at the hospital level had on identifying avoidable gaps.  ***“Ok, like for the MPDSR meetings, which are held at Sub-County level, not within the facility. The Sub-County MOH is the one that determines when these meetings are going to be held. So, we only get an invitation either through SMS, or we get a mail that there is a perinatal meeting that is going to occur on a given date.” FGD 1 (R4)***   1. **Conducted out of obligation as a box-ticking exercise**   Some study participants reported that they regularly held audit meetings at their hospitals. They however expressed their waning confidence in the audit meetings as there were no visible changes that emanated from them. The health workers therefore developed a negative attitude towards the audit process and viewed it as an extra activity that would consume much of their already limited time.  ***“what I don't what I don't like about audits is um... when audit recommendations are not implemented and it's the same… same… things, you know, being reviewed over and over again.”***   1. **The silent “P” in MPDSR**   Perinatal death review was added as a component to the national maternal death surveillance and response (MDSR) guidelines in 2016.[9] The study participants however expressed that the perinatal aspect of the MPDSR was not given as much priority as the maternal component. The MPDSR meetings were scheduled based on the occurrence of a maternal death. In the event that there was none, no meetings were convened to discuss perinatal deaths which were the majority. Contributions from the participants also revealed that there was a normative acceptance of perinatal deaths due to their high volume compared to maternal deaths. This lowered the perceived value placed on auditing individual perinatal deaths.  ***“There's a structure, who's supposed to be the secretary, who is supposed to do ABCDE and it should, you know, this audit should occur within twenty-four hours of… of… of… the maternal death. However, the perinatal actually is disturbing. I think, globally, doctor, all of us will agree, even professor, that it is something we need to strengthen. The "P" aspect of audit, for example in the MPDSR. We need to come up with a structure, really. And I think, currently, I'm not so sure. But I here look at how often do these deaths occur? Maternal audits let's say they happen once. But it doesn't matter, even one maternal audit is a very... I mean death is of great concern. Perinatal audits, people say, from what I've heard from colleagues, in a day, for example for the high-volume hospitals, how many perinatal deaths do occur on a daily basis? So, you are not able, like, to constitute a team immediately, or after 24 hours and discuss. That's why they said... We need actually to be sampling out, but actually, she's brought out it very well. We need to strengthen and come up. I don't know if the… the… team is looking at this, how we need to strengthen the perinatal audits in our facilities.” FGD 3 (R3)***   1. ***Meetings that are not regular or structured***   Some dissonance was noted regarding the scheduling of meetings. Some participants expressed that the audit meetings were frequent and structured while many other participants expressed that audit meetings in their hospitals were not conducted on a regular schedule. The meetings were however dependent on factors such as the availability of the paediatricians and nurse leaders and the attitude of the NBU in-charges towards quality improvement activities.  ***“In our place, the main determinant of these meetings, especially the one in the newborn unit, is the paediatrician, and that's myself. And it depends on my availability, and I usually am the one who has the onus to involve the others, the basic clinical care team that is in the unit” FGD 1 (R6)*** |  |
| 1. **Knowledge to Perform**   The FGD participants brought out that there was a gap between what the health workers should be doing and what they have the knowledge to do. The emerging gaps included: Gaps in problem identification and problem-solving skills, gaps in knowledge due to poor quality of pre-service training, conflicting guidelines and a poor reading culture. | 1. ***Gaps in problem identification and problem-solving skills***   The study participants reported that despite formulating potential solutions to the identified gaps arising during the audit meetings, the same issues kept recurring. The suggested reasons for the recurring issues was due to lack of resources such as medications and materials as the actions suggested required heavy investments that were beyond the scope of the newborn unit health care workers and sometimes beyond the scope of the hospital level management. We noted that the health workers did not have the knowledge to get to the root cause of the problems evidenced by how simple problems were not seen as an issue with focus predominantly on what they did not have. This therefore resulted in the same discussions occurring during every meeting with no change.  ***“Sometimes, like we have, we have had issues with the preterm babies. In our facility, we don't have surfactant factor, we don't have caffeine citrate. So, you realize that it's like the audit, the whole year when we come, it is still caffeine citrate, we don't have, it's still surfactant factor. So, at times, the reason may just recur and recur and recur and recur because of the system, because of the of… of… of… issues to do with the with the procurement.” FGD 3 (R9)***   1. ***Poor quality of pre-service training***   There was an opinion that with every new group of health care worker reporting to the hospitals, there was a decline in the newborn quality of care and an increase in mortality. The group participants expressed that having a new group of trainee clinicians and nurses placed an extra burden on the supervisors as the onus was on them to bring their knowledge up to an acceptable level.  ***“I think sometimes in the department, we'll find people keep on changing like the clinical officers and medical officer interns will keep on coming and we get new ones every other time. So, you may find when they come, they have the same issues. If it's resuscitation, for example, if it's use of CPAP. So, for me, you'll find them recurring, but you see it as a way of knowing where the gaps are and, uh, continuing to teach and, you know, to improve every other time when they come.” FGD 3 (R5)***   1. ***Variation in performance due to conflicting guidelines***   Participants suggested that a significant barrier to providing quality newborn care was the availability of multiple guidelines for the management of the same condition. There were regular updates on patient management guidelines from different groups with no feedback to the frontline health workers on which guidelines should be adapted for patient management. This led to lack of clarity on the management approach they should follow for different conditions and therefore resulting in variation in care for the same conditions.  ***“Now, the problem of the system working, not working. It's also bringing a problem when it comes to the protocol you have introduced. We have the protocol we are all using. We have a new protocol, which is in the internet, and then we have the old protocol. So, there is usually a problem between the dosage and the management, because now the ones who have the new, especially the birth asphyxias. Yes. We have a new management of fluids. So, when you tell them of the protocol, we have, the one of... I think is... the recent one, 2016. So, you see them arguing on the management there. So, we need to have one universal protocol for all of us so that we can be sharing the same information now.” FGD (R7)*** |  |
| 1. **Failure to recognise the complexity of the health system and newborn care**   The contributions from the FGD participants revealed that they recognized that newborn care required team effort. The teams they described were however limited to the immediate newborn care team; nurses and clinicians and occasionally the midwives. Based on this, the perception we got was that the health workers did not fully appreciate the complexity of newborn care based on the collaboration required from various players to ensure its success.  ***“But otherwise, it's just limited to the immediate care team.” FGD 1 (R6)*** |  |  |
| 1. **Knowledge on meaning of clinical audits**   Some contributions in the FGDs revealed that some participants believed that simply reviewing the monthly morbidity and mortality statistics constituted a clinical audit. This was reflected in how the participants responded that the meetings were centred around discussing the newborn statistics that describe the disease and death patterns. We also noted the frequent use of the term ‘morbidity and mortality meetings’ which further reflects that the health workers use these meetings to describe the disease and death patterns in the NBU over a given period.  ***“Yuh, In H4 usually we do combined morbidity, mortality audits together with the Obsgyn and the newborn unit… yuh... Every first Friday of the month. Usually we just look at the statistics.” FGD 2 (R5)*** |  |  |
