# Supplementary material for: Development of a small and sick newborn clinical audit tool and its implementation guide using a human-centred design approach newborn clinical audit process and design
Source: PLOS Glob Public Health. 2023 Feb 23;3(2):e0001577. doi: 10.1371/journal.pgph.0001577 (PMC10021839; doi:10.1371/journal.pgph.0001577)
Supplement: S6 Appendix — (PDF) [file pgph.0001577.s007.pdf]

User Personas

Group one

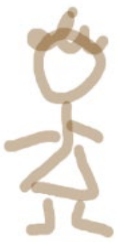

Nurse, Female, Immaculate, 30 years, Married, has 1 child

Neonatal nurse, Diploma holder, 5 Years experience in NBU

Goals: Upgrade to a degree holder in nursing

Roles/responsibilities in NBU:
-Drug administration
-Monitoring babies- temperature, respiration rate etc
-Feeding babies
-Performing neonatal procedures eg photo therapy, CPAP
-Top tailing (cleaning babies)
-Looking after mother of baby
-Giving information on progress and plans for babies to parents
-Record keeping/ documentation
-Supervising other staff
-On night duty covering in hospital
-Assisting students in care of newborns in NBU

Group two

User identifier

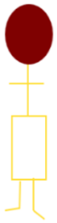

Name: Dr. Mary
Sex: Female
Cadre: General paediatrician
Age: 50years
Duties in NBU: Conducting major ward rounds, chairing the audits, teaching the interns(MOI), NBU incharge of daily running of the unit, managing the staff(eg nurses), member of HMT involved in various processes including budgeting, member of other committees- e.g. infrustructure committee, teaches students (medical stuents and clinical officer students, lecturer at the MTC, works in private hospitals(private practice)
Goals/needs of Dr. Mary: subspecialize in neurology, maintain a work life balance, reduce mortality in the NBU, participate in research in the hospital, increase survival rate in the unit

Group three

NUTRITIONIST

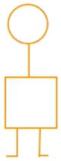

Name: Marion
Sex: Female
Age: 22years old,
Marital status: Single

Responsibilities
- ensure proper attachement and positioning
- monitor growth of the neonate
- ensure required amont of feeds (R1)
- help mother expressed breast milk(R3)
- consel mothers on importance of breast feeding
- ensure mothers are feeding well to provide for the baby(R7)
- to be a team player; find solutions to identified problems and share with other team members, suggesting possible solutions as well(R6)

Goals
- as a fresh graduate: she wants to gather experiences
- looking for a better job(R5)
- to do well and impress her supervisor
- to get a family (R9)
- she wants to do well and get promoted(R7)
- wants to put the knowledge into practice(R1)

experiences
- experience challenges in delivering care (R7)
- being blamed for wrong actions she is scared(R3)
- she finds it strange that what she was taught is not what is in practice
- blamed for being online or having distraction with her phone(R4)
- confused because of working with more grown up people and she is still young with low experience(R5)
- very impatient with mothers and wondering why breat feeding is hard for then
- withholding her ideas
- she feels nutriionist should not be working over weekends(R6)
- she feels she is not being respected being young
- she doesnt know how breast milk is expressed or pumped

Group four

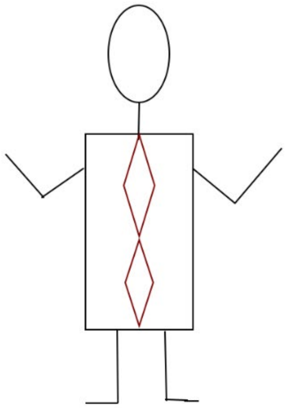

CREATING A USER PERSONA- group 4

Persona- Hospital Administrator
Name: Obala (R9)
Age: 40yrs (R10)
Gender: Female
Tasks: - doing a management round esp if visitors are expected
- deal with complaints from patients and issues such as waivers
- Coordinates transport eg the ambulance has fuel
- R5- ensuring utility bills are paid and machines are serviced
- managing funds for food and linda mama
-R9- office work eg liason with county officials
- R10- involved in budgetary meetings, ensure utilisation is achieved on what allocated
Goals:
- hospital budget-
-

work experience: good and probably motivated
frustrations faced:- may get overwhelmed with the duties/ tasks
- frequent changes in office occupancy (politically driven)

Additional factors by "Obala"
- staff capacity/ training eg are there neonatal nurses?
- Mothers and how they are housed
- staff motivation

Community health workers/ community level issues- are they a separate category
