# Supplementary material for: Development of a small and sick newborn clinical audit tool and its implementation guide using a human-centred design approach newborn clinical audit process and design
Source: PLOS Glob Public Health. 2023 Feb 23;3(2):e0001577. doi: 10.1371/journal.pgph.0001577 (PMC10021839; doi:10.1371/journal.pgph.0001577)
Supplement: Attachment — Submitted filename: Response to Reviewers.docx [file pgph.0001577.s009.docx]

**Ref: Submission ID PGPH-D-22-00756R1,**

**Editorial board,**

**PLOS Global Public Health,**

**12^th^ December 2022.**

**Re: “Development of a small and sick newborn clinical audit tool and its implementation guide using a human-centred design approach”**

We thank the editorial board members and reviewers for taking the time to review this manuscript. We acknowledge receipt of the comments made and we feel they have improved the value and quality of the manuscript. These are the responses and changes made based on the comments.

**Journal requirements**

**We have noticed that you have uploaded "Supporting Data" in Supporting Information files, but you have not included a list of legends. Please add a full list of legends for your Supporting Information files after the references list.**

**Thank you for this comment.**

We have included the legend on supporting data in lines 660 -664.

“We have provided the supporting data as files and these include:

1. The audit prototype tools for each stage of the cognitive walkthrough.
2. The consensus workshop data.
3. The data from the focus group discussions.”

**Reviewer one comments**

1. **The part of the methods section describing study setting / participants is very long and would benefit from sub-headings to sign-post/guide the reader, linked to the phases of the study. The description of study sites is very long and the article would benefit from this section being more concise.**

Thank you for the comments. We have addressed them as follows:

We have placed subheadings linking the study setting to the phase of the study as outlined on lines 100 and 109. We have also edited this section and focused on describing the key details that would impact the study – lines 100 – 117.

**Description of study sites for the design of the audit tool**

“Pumwani Maternity Hospital (PMH) is one of the County referral hospitals in Kenya and was selected as the site for the design of the SSNB audit tool.[22] This hospital was selected as at the time of the study, the lead researcher (MO) had been a paediatrician in the hospital for approximately one year. It is also the largest referral maternity hospital in Kenya located in Nairobi County with approximately 100 deliveries per day.[23] The NBU admits approximately 350-400 newborns per month and provides intermediate-level care.[24] The hospital held monthly MPDSR meetings to discuss maternal near misses with a brief overview of perinatal morbidity and mortality statistics. In the event of a maternal death, the meeting was held within 48 hours of the death.”

**Description of study sites for usability testing**

The initial plan was to test the tools in PMH. However, Kenyatta National Hospital (KNH), the largest teaching and referral hospital in Kenya expressed interest in improving its newborn clinical audit process and was incorporated into the study as a testing site in January 2021. KNH caters for low-and-middle-income populations from Nairobi and its environs as well as referrals from other hospitals in the country and the greater Eastern Africa region.[25] The NBU of KNH admits approximately 250 – 300 newborns per month and offers intensive care to critically ill newborns.[24] The NBU staff do not attend MPDSR meetings, they however infrequently hold in-house meetings to discuss the NBU morbidity and mortality statistics.

2. **Box 2 – It would be helpful to have the numbers of each participant type in the box, for ease of access by readers.**

Thank you for the suggestion.

The participants summarised in box 2 are those involved in the cognitive walkthrough and usability testing while the ones with numbers are those involved in the virtual workshop. To make this clear, we have put sub-headings highlighting the participants involved in each phase on lines 119 and 122.

**“Cognitive walkthrough and usability testing of prototype audit tools in the hospitals**

The SSNB audit tool was intended for use by a transdisciplinary team who directly provide care to the SSNB and were therefore involved in its design and testing its usability (Box 2).

**Virtual design workshop**

The design workshop of the implementation guide involved 37 purposely selected participants (two neonatologists, 17 NBU nurse leaders, 17 paediatricians and an MoH official) and was held using the Zoom platform.”

3. **In general, the methods are very long and contain very detailed and extraneous information which could be removed, to make the manuscript more concise and readable:
   E.g. detailing the day of the week when meetings were held is not necessary, instead it is sufficient to state that meetings were initially held monthly then every two-weeks (lines 174 – 177). Detailing who prepared the meetings is not necessary in the text, as this is outlined in Box 3. I suggest the authors review the methods section and edit the word count down where possible, using clearly labelled sub-headings.**

Thank you for the suggestions.

To address this, we have edited the methods section by editing out the suggested lines.

We have also made the methods section more concise by focusing on the methods used in the three phases – design of the audit tool (draft zero and the subsequent prototypes), design of the audit implementation guide and the usability testing of the two.

4. **This is a complicated mixed methods study with multiple components occurring at different timepoints. A figure giving an overview of the activities and timelines would be very helpful for the reader to appreciate how long it took and how the different components overlapped temporally.**

Thank you for the suggestion.

We have addressed this by including the time points for each phase in figure 1 which describes the three study phases.

5. **The authors state that a focus group discussion was conducted. More information about this qualitative method is required - particularly who conducted the FGD, what was the intended sample size for the FGD and how were participants selected, how was the data analysed, any methods used to enhance the trustworthiness & reliability of the data.**

Thank you for the comment.

We have addressed this by including information on who conducted the FGDs, number of respondents and how they were selected , data analysis and methods used to enhance the reliability of the data in supplementary file 1 as summarised below:

“We conducted FGDs with the 37 workshop participants. A purposive sampling strategy was used to divide the participants into four groups using Zoom breakout rooms with a sample size of 9-10 each. The purposive sampling ensured there was an equal number of nurses and paediatricians in each group. Each of the four groups had:

1. A moderator who was either a nurse or a paediatrician who had group facilitation skills as they had gone through the UK Resuscitation’s Council Generic Instructor’s Course (GIC) in Kenya.[29]
2. A rapporteur who was a researcher from KWTRP who had experience in conducting qualitative research and moderating small groups.
3. A note taker who was a paediatric resident from UoN, was GIC trained and was proficient in using the Zoom platform and its different features. The rapporteur helped to manage group dynamics and the note taker helped to document responses on the virtual whiteboard.

Each group moderator had a discussion guide with open-ended questions to help structure the discussions and each group had a different set of questions. The discussion guide also contained probing questions to help the moderator cover issues in depth with the less talkative groups. Consent to participate in the FGDs was obtained through a two-step process; i) An electronic version of the consent forms was emailed and sent via WhatsApp to each invited participant three days before the workshop. They were requested to sign the form or fill in their names and send it back to the main facilitator (MO) before the workshop if they agreed to participate in the workshop, ii) before beginning the FGDs, each moderator projected the consent form and a confidentiality agreement statement and read them out to the group members. The participants were then asked to directly message MO via Zoom chat with the statement “I agree” or unmute and confirm that they agree if they agreed to participate. Data collection was done through audio recording as well as the information documented on the whiteboard.

**Data analysis**

The data were managed on NVivo 12 software. The data analysis was done through thematic analysis. The audio-recorded data from the FGDs were transcribed verbatim. After transcription, the first step was familiarisation with the data by reading through it severally. Two researchers, MO and GI independently studied the transcripts and independently generated the initial codes. An abstraction process then took place and the codes were grouped into themes. The entire analytic process involved collaboration with MO and GI, and all discrepancies were discussed until consensus was reached.”

6. **Line 232 – verbal or written consent was obtained prior to the FGDs – this is slightly unusual. In which cases were verbal consent obtained and why not always written consent?**

Thank you for the question.

We have clarified that an explanation of the study and the components of the workshop including the FGD were sent to the participants via email and they consented to participate in all the components of the workshop. Consent was however obtained again before the FGD for extra precaution and the participants consented to participate by sending a direct message “I agree” to the group moderator. Due to some challenges with participants who were not proficient with the Zoom platform, we accepted that they could unmute and say “I agree” to give their consent. (lines 219 – 223).

“The participants signed an electronic consent form and emailed it to the lead researcher (MO) three days before the workshop (S2 appendix). In addition, the participants gave consent again before the FGD by directly messaging the group moderator “I agree”, there were however a few participants who were not proficient in using the Zoom platform and were allowed to unmute and give their verbal consent.”

7. **The FGD findings are presented halfway through the results section and may be lost to the reader. I would suggest that the authors consider making these findings more prominent in the manuscript, as there is rich learning from this part of the study around facilitators/barriers.**

Thank you for this comment.

To make the FGD results more prominent, I have expounded on the statement directing readers to the supplementary appendix S5 where the results are highlighted. Line 295.

8. **Table 2 contains useful, practical information for other centres wanting to do similar work.**

We thank you for this comment.

9. **Lines 354 – 358: This level of detail about some members of the audit committee not attending all meetings is not required. Consider making this paragraph more concise and focus on the roles of the committee members instead.**

Thank you for this suggestion.

We have addressed this by editing this paragraph and focusing on the important details such as the role of the committee members (lines 338 – 339).

10. **Lines 366 – 387: It is not clear to me how this information is a finding of the study? This may be better suited to being in the discussion section.**

Thank you for this comment.

Part of this explanation has been included in the discussion (lines 380 – 382).

“The use of real cases however led to several interruptions in the design process due to several HCW strikes and restrictions on meetings due to the COVID-19 pandemic.”

11. **The first paragraph of the discussion section is well written and summarises the findings / work nicely. However, the rest of the discussion is very long and would benefit from being shorter and focusing only on key findings and learning with clear sub-headings relating to the objectives of the study.**

Thank you for the comment.

We have made the discussion section more concise by further summarising all the paragraphs in the discussion section as seen in lines 373 – 458.

We have also clearly assigned sub-headings highlighting the results being discussed based on the phase of the study.

**In summary, this manuscript has the potential to add to the literature for this topic but requires more work to clearly present the methods and findings in a coherent and concise way. In addition, more information about the methods of the qualitative aspect (FGDs) is required.**

**Reviewer two comments**

a) **Abstract is well written but line 31, the authors have used abbreviation (NBUs) without defining it. I suggest writing NBUs in full in the abstract.**

Thank you for this observation.

We have written NBUs in full “newborn units” in the abstract – line 31

b) **Introduction is also well written, but to make it clearer, few suggestions should be considered.
   Line 40, majority of deaths-add the figures to quantify the burden**

Thank you for this suggestion.

We have included the figures in line 41 – 82%. **Line-69-70. You have introduced HCD well and mentioned how it is used,
Please elaborate more on what is HCD approach, and rationale for its use in clinical audits.**

Thank you for this comment.

We have expounded on what HCD approach is, and included an explanation on why a HCD approach was considered appropriate for the design of the clinical audit tool in lines 72 – 79.

“This “is a design approach that puts human needs, capabilities, and behaviour first, then designs to accommodate those needs, capabilities, and ways of behaving.”[13] This promotes the development of usable systems by advocating for active user participation and allowing for several iterations of a design of the system and subsequent modifications based on the users’ requirements.[16, 17] A HCD approach was considered appropriate for the design of a context sensitive clinical audit tool and implementation guide as it presumes that the users of an innovation understand its core challenges, and therefore hold the key to its solution.[18]”

3. **Methods section
   a) Line 117- What criteria/s did you use to choose PMH to test usability of the
   audit tool?**

Thank you for this question.

“We have elaborated on the method of selection for PMH in lines 101 – 106.

Pumwani Maternity Hospital (PMH) is one of the County referral hospitals in Kenya and was selected as the site for the design of the SSNB audit tool . This hospital was selected as at the time of the study, the lead researcher (MO) had been a paediatrician in the hospital for approximately one year. It is also the largest referral maternity hospital in Kenya located in Nairobi County with approximately 100 deliveries per day.” **b) It’s not clear if the tool was meant for auditing deaths, or neonatal cases or both, please clarify**

Thank you for this question.

We have explained that the audit tool was designed to audit the quality of care provided to newborns who died and those who survived but had a near miss event in lines 65 – 67. **c) You used consensus meetings and FGDs as some of methodologies- elaborate more on who was involved in FGDs, sampling and how you analysed FGD data**

Thank you for the comment.

We have addressed this by including information on who conducted the FGDs, the sample size, the selection of participants, data analysis and methods used to enhance the reliability of the data in supplementary file 1 as summarised below:

“We conducted FGDs with the 37 workshop participants. A purposive sampling strategy was used to divide the participants into four groups using Zoom breakout rooms with a sample size of 9-10 each. The purposive sampling ensured there was an equal number of nurses and paediatricians in each group. Each of the four groups had:

1. A moderator who was either a nurse or a paediatrician who had group facilitation skills as they had gone through the UK Resuscitation’s Council Generic Instructor’s Course (GIC) in Kenya.[29]
2. A rapporteur who was a researcher from KWTRP who had experience in conducting qualitative research and moderating small groups.
3. A note taker who was a paediatric resident from UoN, was GIC trained and was proficient in using the Zoom platform and its different features. The rapporteur helped to manage group dynamics and the note taker helped to document responses on the virtual whiteboard.

Each group moderator had a discussion guide with open-ended questions to help structure the discussions and each group had a different set of questions. The discussion guide also contained probing questions to help the moderator cover issues in depth with the less talkative groups. Consent to participate in the FGDs was obtained through a two-step process; i) An electronic version of the consent forms was emailed and sent via WhatsApp to each invited participant three days before the workshop. They were requested to sign the form or fill in their names and send it back to the main facilitator (MO) before the workshop if they agreed to participate in the workshop, ii) before beginning the FGDs, each moderator projected the consent form and a confidentiality agreement statement and read them out to the group members. The participants were then asked to directly message MO via Zoom chat with the statement “I agree” or unmute and confirm that they agree if they agreed to participate. Data collection was done through audio recording as well as the information documented on the whiteboard.

**Data analysis**

The data were managed on NVivo 12 software. The data analysis was done through thematic analysis. The audio-recorded data from the FGDs were transcribed verbatim. After transcription, the first step was familiarisation with the data by reading through it severally. Two researchers, MO and GI independently studied the transcripts and independently generated the initial codes. An abstraction process then took place and the codes were grouped into themes. The entire analytic process involved collaboration with MO and GI, and all discrepancies were discussed until consensus was reached.”

4. **Results
   a) Table one- good way of showing what is available on the ground but it could be better if the new tool developed in this study could be also compared against what is currently available to show uniqueness of the tool developed in this study in Table 1**

Thank you for the suggestion.

We have included box 4 which highlights the differences and additions in the co-designed clinical audit tool compared to the other tools summarised in tale 1 – line 307.

| **Box 4: Differences between the co-designed SSNB audit tool and the available audit tools** | | |
| --- | --- | --- |
| **Design details** | **Structure of co-designed audit tool** | **Structure of available audit tools** |
| 1. **Sections of the audit tools.** | - The audit tool has two sections. The first section is to provide a summary of the care provided. The second section is a guide for documenting the discussion on the quality of care in each section. | - Only the WHO child and neonatal death review form has provision to document the gaps identified during the audit meeting discussion. The discussion however comes after each section describing the summary of care. |
| 2. **Structure of input fields.** | - The basic structure mostly includes check boxes, drop-down calendars and open-ended questions. | - The MPDSR tool and the WHO stillbirth and neonatal death case review form have fields structured as closed-ended questions.   The WHO child and neonatal death review form is structured as open-ended questions with free text.  None of the tools have checkboxes and drop-down calendars. |
| 3. **Resuscitation and post-resuscitation care.** | - The section on newborn resuscitation immediately after delivery specifies the type of resuscitation provided and includes the post-resuscitation care provided. | - The available audit tools only document if the newborn was resuscitated but do not provide details of the resuscitation. They do not provide details on post-resuscitation care. |
| 4. **Details of admission.** | - The details of admission include details on delays in transfer from the delivery unit to the NBU and delays in the review of the newborn by the clinician while in the NBU. | - The available audit tools do not include details on the delays in transfer of the newborn to the NBU and the delays in review of the newborn during admission. |
| 5. **Description of clinical illness and progression.** | - A section to comprehensively describe the danger signs at admission and a different section to describe the progression of clinical illness post-admission. | - The WHO child and neonatal death review form include a section to summarise the child’s illness and progression in two lines. |
| 6. **Nursing care audit.** | - A section to audit the nursing care provided during the hospital stay. | - None of the available audit tools have provisions to audit the nursing care provided to the newborns. |
| 7. **Treatment provided.** | - Detailed description of the supportive and definitive treatment provided to the newborn including the feed and fluid management. | - The WHO child and neonatal death review form include a section to describe the treatment provided. This is however not grouped into supportive and definitive treatment. |
| 8. **Action plans.** | - A structured action plan summary form that is part of the audit tool. | - The WHO child and neonatal death review form include a structured action plan summary form that is separate from the audit tool. - The other audit tools have a free text section to list down the action plans. |
| 9. **Audit meeting attendees.** | - A list with a check box to identify the members of the audit committee who attended the audit meeting. | - None of the available audit tools have a section to document the audit meeting attendees. |
| 10. **Modifiable factors.** | - Structured list of modifiable factors categorised into administrative-related, health worker related and patient oriented factors. These have check boxes for selection instead of free text. | - The MPDSR tool has a list with a checkbox for selection. Categorised as the three delays. - The WHO child and neonatal death review form has a list that is categorised as family, administrative and provider related modifiable factors. - The WHO stillbirth and neonatal death case review form has a free text section to list down identified modifiable factors. |

**b) When describing the outcome of available tools on line 248-251, you have described about gaps with Kenya MPDSR tool, WHO stillbirth and neonatal tool and paediatric audit tool. There is mention of perinatal as well as neonates. Definition of perinatal, neonates and its cut off age would assist to make clear where gap is interns of tools that are already in existence and the one you have developed.**

Thank you for the comment**.**

We have defined the perinatal and neonatal periods in lines 53 – 55.

“The perinatal period, as defined by the WHO, “commences at 22 completed weeks of gestation and ends at seven completed days after birth.”[11] The neonatal period commences at birth and ends at 28 completed days after birth.” **c) On FGDs- you have not included participants quotes to support your summary of findings. I have seen quotes in supporting information appendix 5. Include a statement for readers to refer to participants quotes in supplementary information when reading the summary of FGDs findings**

Thank you for this suggestion.

We have included a statement referring readers to supporting information to read participant quotes in line 295.

**Strengths and limitation
a) As indicated you only included 2 hospitals- How are the findings applicable to other facilities? Would you please clarify generalisability of findings in your limitation/strength section**
Thank you for the comment.

We have included a statement on the generalisability of the study findings in lines 472 – 475.

“The two hospitals represented a high-volume county referral hospital and a tertiary level hospital. This may therefore not be generalisable to lower level hospitals which may not have the capacity to implement the implementation guide as designed.”

Again, I thank all the reviewers for the useful input to this manuscript.

Yours Sincerely,

Dr. Muthoni Ogola.
